# Heterogeneous Role of the Glutathione Antioxidant System in Modulating the Response of ESFT to Fenretinide in Normoxia and Hypoxia

**DOI:** 10.1371/journal.pone.0028558

**Published:** 2011-12-08

**Authors:** Tapiwanashe Magwere, Susan A. Burchill

**Affiliations:** Leeds Institute of Molecular Medicine (LIMM), Children's Cancer Research Group, St. James's University Hospital, Leeds, United Kingdom; Biological Research Center of the Hungarian Academy of Sciences, Hungary

## Abstract

Glutathione (GSH) is implicated in drug resistance mechanisms of several cancers and is a key regulator of cell death pathways within cells. We studied Ewing's sarcoma family of tumours (ESFT) cell lines and three mechanistically distinct anticancer agents (fenretinide, doxorubicin, and vincristine) to investigate whether the GSH antioxidant system is involved in the reduced sensitivity to these chemotherapeutic agents in hypoxia. Cell viability and death were assessed by the trypan blue exclusion assay and annexin V-PI staining, respectively. Hypoxia significantly decreased the sensitivity of all ESFT cell lines to fenretinide-induced death, whereas the effect of doxorubicin or vincristine was marginal and cell-line-specific. The response of the GSH antioxidant system in ESFT cell lines to hypoxia was variable and also cell-line-specific, although the level of GSH appeared to be most dependent on *de novo* biosynthesis rather than recycling. RNAi-mediated knockdown of key GSH regulatory enzymes γ-glutamylcysteine synthetase or glutathione disulfide reductase partially reversed the hypoxia-induced resistance to fenretinide, and increasing GSH levels using *N*-acetylcysteine augmented the hypoxia-induced resistance in a cell line-specific manner. These observations are consistent with the conclusion that the role of the GSH antioxidant system in modulating the sensitivity of ESFT cells to fenretinide is heterogeneous depending on environment and cell type. This is likely to limit the value of targeting GSH as a therapeutic strategy to overcome hypoxia-induced drug resistance in ESFT. Whether targeting the GSH antioxidant system in conjunction with other therapeutics may benefit some patients with ESFT remains to be seen.

## Introduction

Glutathione (γ-glutamylcysteinylglycine, GSH) is the most abundant non-protein thiol in mammalian cells that functions both as a homeostatic redox buffer and in cellular defense against free radicals and reactive electrophiles [1], [2]. In cancer cells, GSH has a dual role both in preventing the initiation of cancer and promoting the progression of disease through GSH-dependent drug resistance mechanisms [2], [3], [4]. Consequently, anticancer strategies targeting the GSH redox system such as *redox chemotherapy* have been developed to modulate the intracellular redox environment with the expectation this would sensitize cancer cells to therapy [5] leading to improved therapeutic response and outcome.

Although redox chemotherapy is an emerging anticancer strategy, it has not been as successful in the clinic as preclinical studies indicated it might [6], [7]. We propose that this may reflect the presence of hypoxic regions in some tumours that confer resistance to apoptosis and increase the metastatic potential of malignant cells as previously reported [8], [9]. Hypoxia-induced drug resistance mechanisms have been widely described [10], [11], [12], but the molecular mechanisms are not fully resolved. In this study we have hypothesised that hypoxia may cause up-regulation of the GSH antioxidant system in ESFT thereby attenuating the efficacy of some therapeutics.

Three key enzymes are involved in the regulation of cellular GSH at the level of biosynthesis (γ-glutamylcysteine synthetase, GCS), regeneration/recycling (glutathione disulfide reductase, GRD) and breakdown (γ-glutamyltranspeptidase, GGT). GCS is the initiating and rate-limiting enzyme in the biosynthesis of GSH within cells, whereas GRD and GGT are involved in the regeneration and breakdown respectively of oxidized GSH (glutathione disulfide, GSSG). GCS is the target enzyme for inhibition by the GSH-depleting agent *L*-buthionine S, R-sulfoximine (BSO) [13] which has been exploited to sensitize some cancer cells to cytotoxic drugs such as melphalan and cisplatin [14], [15]. However GGT might also be a valuable therapeutic target because it is up-regulated in many aggressive cancers where it is important in the maintenance of cellular GSH redox homeostasis [16].

Ewing's sarcoma is the second most common bone malignancy found in children and young adults [17], and belongs to the Ewing's sarcoma family of tumours (ESFT). ESFT are characterised by non-random gene rearrangements between the *EWS* gene on chromosome 22q12 and an *ETS* gene family member; the *t*(11;22)(q24;q12) chromosome rearrangement that produces the chimeric EWS/Fli1 fusion gene is the most frequently described. These pathognomonic gene fusions are major drivers of the development and maintenance of the ESFT malignant phenotype [17], [18]. Current treatment for patients with metastatic ESFT includes a combination of surgery, radiotherapy and chemotherapy, incorporating doxorubicin, vincristine, cyclophosphamide, and etoposide [19]. Despite this intensive treatment regime, the 5 year survival rate for patients with metastatic disease at diagnosis is less than 25% [20], prompting the need for new treatment approaches for this cancer. Fenretinide (*N*-(4-hydroxyphenyl)-retinamide) is a synthetic retinoic acid derivative that has documented cytotoxicity in a wide variety of malignant cell types [21], [22], [23], including ESFT [10], [24], [25]. Fenretinide induces cell death in malignant cells through overproduction of ROS that is thought to activate the intrinsic cell death cascade [22], [24]. This mechanism of action and minimal toxicity in both adults [26], [27] and children [28], [29] makes fenretinide an attractive candidate for the potential treatment of ESFT.

Previous studies from our laboratory have demonstrated that fenretinide kills ESFT cells *in vitro* at concentrations achievable *in vivo* [24], [25]. Furthermore we have identified the cellular antioxidant GSH as a determinant of the sensitivity of ESFT cells to fenretinide in normoxia [25]. Whether GSH plays a similar role in hypoxia-induced drug resistance was the major question for this study. We therefore investigated the effect of hypoxia on GSH and its regulatory enzymes and the response of ESFT cells to selected therapeutics including fenretinide in normoxia and hypoxia. The results presented here demonstrate that the involvement of GSH in hypoxia-induced drug resistance in ESFT is cell line-specific, and might therefore be tumour-specific. These observations suggest that optimal patient benefit from redox-based therapies targeting the GSH antioxidant system may depend on patient-specific tumour characteristics.

## Results

### ESFT cells are less sensitive to selected chemotherapeutic agents in hypoxia

The overall aim of these experiments was to determine whether the culture environment (normoxia or hypoxia) influenced the effect of fenretinide, doxorubicin, and vincristine on ESFT viable cell number. Stabilization of GLUT1 protein confirmed the hypoxic culture conditions in these experiments (Supporting Information S1). All five ESFT cell lines were statistically significantly less sensitive to fenretinide in hypoxia compared to normoxia (Table 1). In contrast, only one of the five cell lines (TC-32) showed decreased sensitivity to doxorubicin, and 3 (TC-32, RDES and SKES-1) out of 5 showed decreased sensitivity to vincristine in hypoxia. The effect of hypoxia on the IC-50 values for doxorubicin and vincristine was marginal (p≤0.05) compared to its effect on response to fenretinide (Table 1). These results suggest that hypoxia-mediated resistance of ESFT cells to chemotherapeutic agents could be more relevant when these agents initiate cell death through production of ROS as is the case with fenretinide.

**Table 1:** The IC-50 values^a^ for chemotherapeutic agents in ESFT cell lines incubated with indicated agents for 48 hours in normoxia and hypoxia.

Fenretinide (µM)			
*Cell Line*	*Normoxia*	*Hypoxia*	*P-value*
A673	0.42 (0.25–0.59)	3.97 (2.50–5.44)	<0.001
RDES	0.59 (0.24–0.84)	1.69 (1.39–1.99)	<0.01
SKES-1	0.81 (0.33–1.29)	12.6 (10.6–14.9)	<0.001
TC-32	0.75 (0.44–1.06)	7.22 (4.94–9.50)	<0.001
TTC-466	0.25 (0.11–0.39)	1.18 (0.40–1.96)	<0.05

Doxorubicin (nM)			
*Cell Line*	*Normoxia*	*Hypoxia*	*P-value*
A673	22.6 (18.0–30.0)	34.6 (26.8–49.9)	*NS*
RDES	23.1 (19.4–29.0)	15.9 (13.5–18.9)	*NS*
SKES-1	12.6 (10.5–14.9)	12.6 (10.6–14.9)	*NS*
TC-32	4.3 (3.6–5.0)	6.1 (5.3–7.2)	<0.05
TTC-466	24.7 (20.8–31.2)	17.3 (15.2–19.8)	*NS*

Vincristine (nM)			
*Cell Line*	*Normoxia*	*Hypoxia*	*P-value*
A673	3.2 (2.7–3.7)	4.0 (3.4–4.7)	*NS*
RDES	4.6 (4.2–5.1)	5.6 (5.1–6.3)	<0.05
SKES-1	3.7 (3.2–4.1)	4.7 (4.1–5.5)	<0.05
TC-32	6.0 (5.2–6.8)	9.0 (8.0–10.3)	<0.05
TTC-466	3.1 (2.7–3.5)	5.2 (4.6–6.1)	*NS*

a IC-50 values represent the drug concentration that reduced viable cell number by 50% as assessed by the trypan blue exclusion assay. Values given are IC-50 value (lower-upper 95% CI) for six replicates from 2 independent experiments. *NS*: Difference not statistically significant (*p>0.05*).

### Fenretinide-dependent decrease in viable cell number is effected through induction of apoptosis, which is decreased by hypoxia

**Figure 1:**
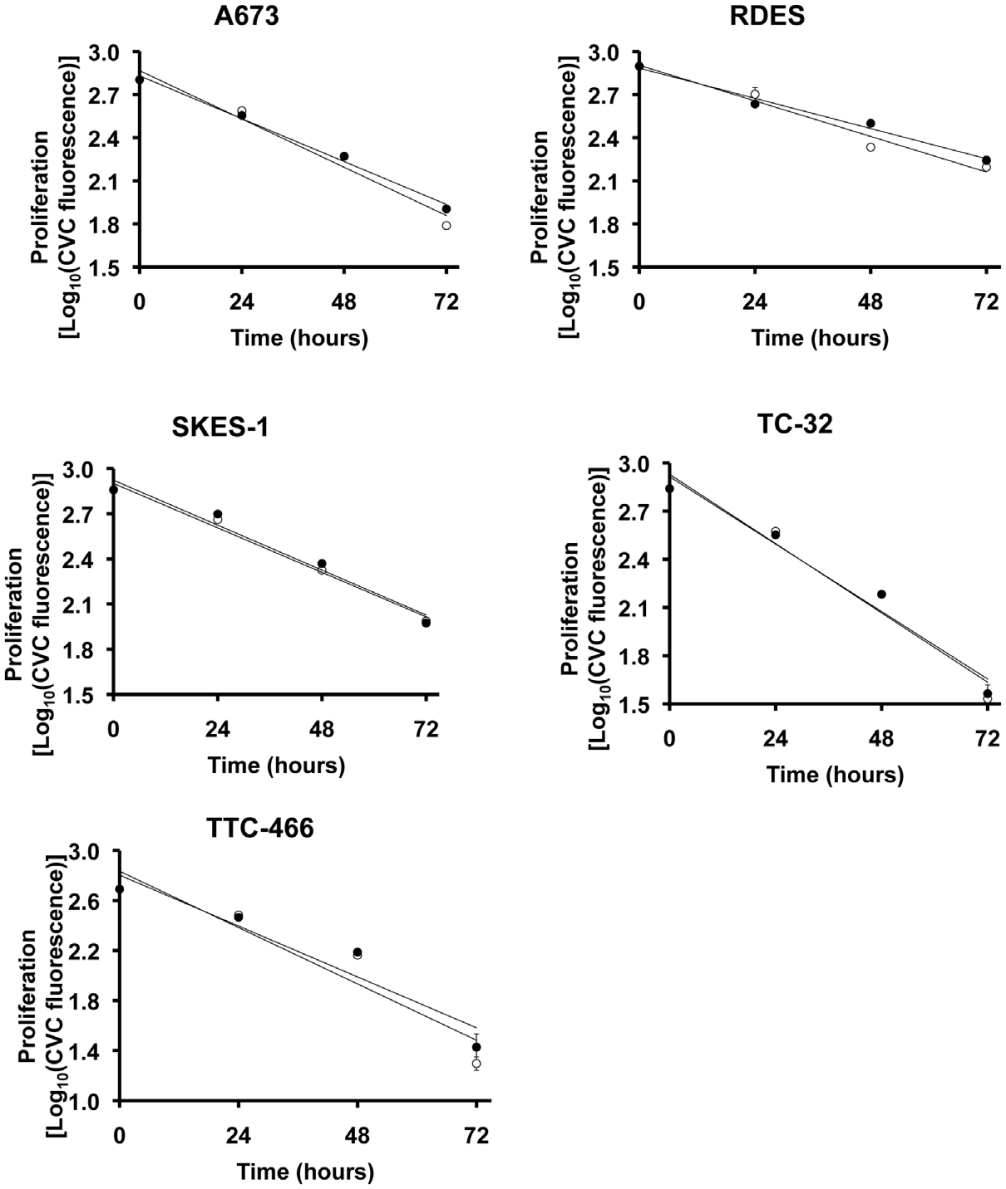
The effect of hypoxia on proliferation of ESFT cells. Proliferation was measured as the decrease in fluorescence of the plasma membrane-labelling dye CellVue™ Claret (see text for details). The graphs are semi-log plots of log_10_ (CVC fluorescence) *vs*. time, and differences between slopes were compared by linear regression analysis. Values represent the mean ± SEM of n = 6 determinations. Open circles = normoxia, closed circles = hypoxia.

Having demonstrated that the cytotoxicity of fenretinide on ESFT cells was reduced by hypoxia, we went on to investigate whether this reflected hypoxia-induced changes in proliferation or apoptosis. Previous studies have suggested in some cell types that the effect of fenretinide may in part be due to fenretinide-induced decrease in proliferation [30]. However our results showed that proliferation of all 5 ESFT cell lines was unaffected by either hypoxia (Figure 1) or fenretinide treatment (Table 2). There was a significant reduction in the level of apoptosis in ESFT cells incubated with fenretinide in hypoxia (Table 2 and Supporting Information S1), demonstrating that the decrease in fenretinide-induced cell number in hypoxia reflected a decrease in apoptosis. Hence from these results we can conclude that fenretinide decreased ESFT viable cell number through induction of apoptosis, which is reduced by hypoxia. The effects of doxorubicin and vincristine on proliferation were cell line specific, in the majority of cell lines proliferation was unchanged (Supporting Information S1). The effect of hypoxia on the induction of apoptosis by doxorubicin or vincristine was also heterogeneous (Supporting Information S1).

**Table 2:** Effects of fenretinide on proliferation and apoptosis levels of ESFT cells in normoxia and hypoxia over 48 hours.

*Proliferation (Slope of linear regression curve)* a			
Cell Line	Normoxia	Hypoxia	P-value
A673	−0.0113±0.0005	−0.0112±0.0002	*0.7264*
RDES	−0.0085±0.0003	−0.0079±0.0002	*0.0910*
SKES-1	−0.0101±0.0005	−0.0095±0.0006	*0.5132*
TC-32	−0.0076±0.0004	−0.0077±0.0006	*0.9159*
TTC-466	−0.0100±0.0004	−0.0102±0.0003	*0.7187*

*Apoptosis (% apoptotic cells)* b			
Cell Line	Normoxia	Hypoxia	P-value
A673	45.3±1.3	15.0±1.0	0.0022*
RDES	72.1±2.3	26.3±2.0	0.0022*
SKES-1	57.4±1.3	24.0±3.0	0.0022*
TC-32	55.0±2.3	22.2±1.7	0.0022*
TTC-466	78.3±3.4	43.8±2.4	0.0022*

a This is the slope of the graph of log CVC fluorescence *vs*. time. A full description of the analyses is given in the text.

b Apoptosis was assessed by Annexin-V/PI staining, acquired by FACS and analysed using CellquestPro™ software. The percentage value shown is the sum total of cells that stained positive for Annexin-V alone, Annexin-V+PI together, and PI alone. The data were compared statistically using the non-parametric Mann-Whitney-Wilcoxon rank sum test. Values shown are mean ± SEM of n = 6 determinations.

*Value in hypoxia is significantly different from normoxia at *p* value indicated.

### Hypoxia does not alter fenretinide-induced ROS in ESFT cells

**Figure 2:**
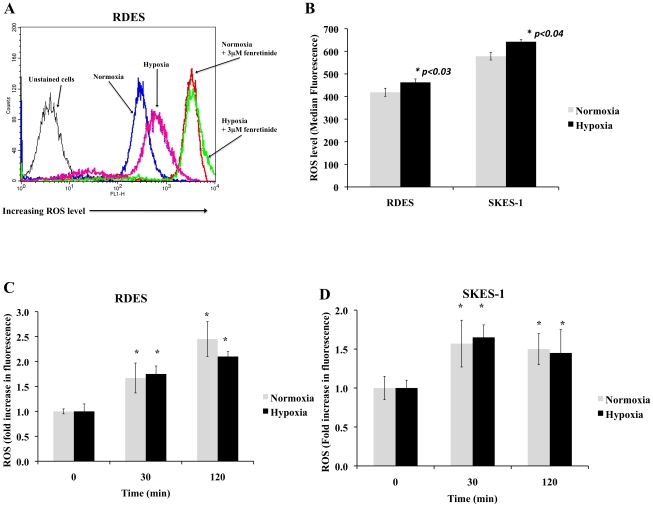
Fenretinide induces ROS production in both normoxia and hypoxia. (A) Representative histograms of the effects of hypoxia and treatment with fenretinide on ROS production in the RDES cell line. (B) Hypoxia caused a significant increase in basal levels of ROS in both RDES and SKES-1 cell lines in the absence of fenretinide. (C–D) The magnitudes of ROS increase following treatment of ESFT cells with fenretinide (3 µM) were similar in normoxia and hypoxia over a period of 30 min–2 hrs. Values are mean ± SEM of n = 3 independent experiments. *Value significantly different from zero time control at p≤0.01.

Previous studies have shown that fenretinide-induced cell death in ESFT cells is dependent on increased level of ROS in normoxia [24]. We therefore selected two cell lines, RDES and SKES-1, to establish if the decrease in apoptosis of ESFT cells in hypoxia was due to alteration in increased level of ROS. Cells cultured in hypoxia for 12 hours in the absence of fenretinide had a significant increase in basal ROS levels compared to those cultured in normoxia (Figure 2A & B). However, when these cells were treated with 3 µM fenretinide for 30 min–2 hrs (Figure 2C and 2D), a much larger increase in ROS levels was observed but there was no significant difference in the magnitude of ROS increase between cells incubated with fenretinide in hypoxia compared to normoxia. These results demonstrate that hypoxia does not affect the ability of fenretinide to induce ROS, and suggest that ROS may not be a critical regulator of the death cascade in these ESFT cells.

### The effects of hypoxia on GSH levels and GSH-regulatory enzymes were cell-line-specific

Since GSH is the major intracellular redox buffer that we have previously shown modulates the sensitivity of ESFT cells to fenretinide in normoxia [25], we investigated the hypothesis that GSH and its regulatory enzymes may be responsible for the reduced sensitivity of ESFT cells to fenretinide in hypoxia. In order to investigate the putative role of GSH in hypoxia-induced resistance mechanisms while minimizing the influence of different ESFT genotypes, we selected two cells lines (RDES and SKES-1) with the same EWS/Fli1 fusion type for further study; both cell lines contain the EWS/Fli1 type II fusion and express p53 and p16. The results for the other 3 ESFT cell lines are presented in Supporting Information S1.

#### GSH levels

**Figure 3:**
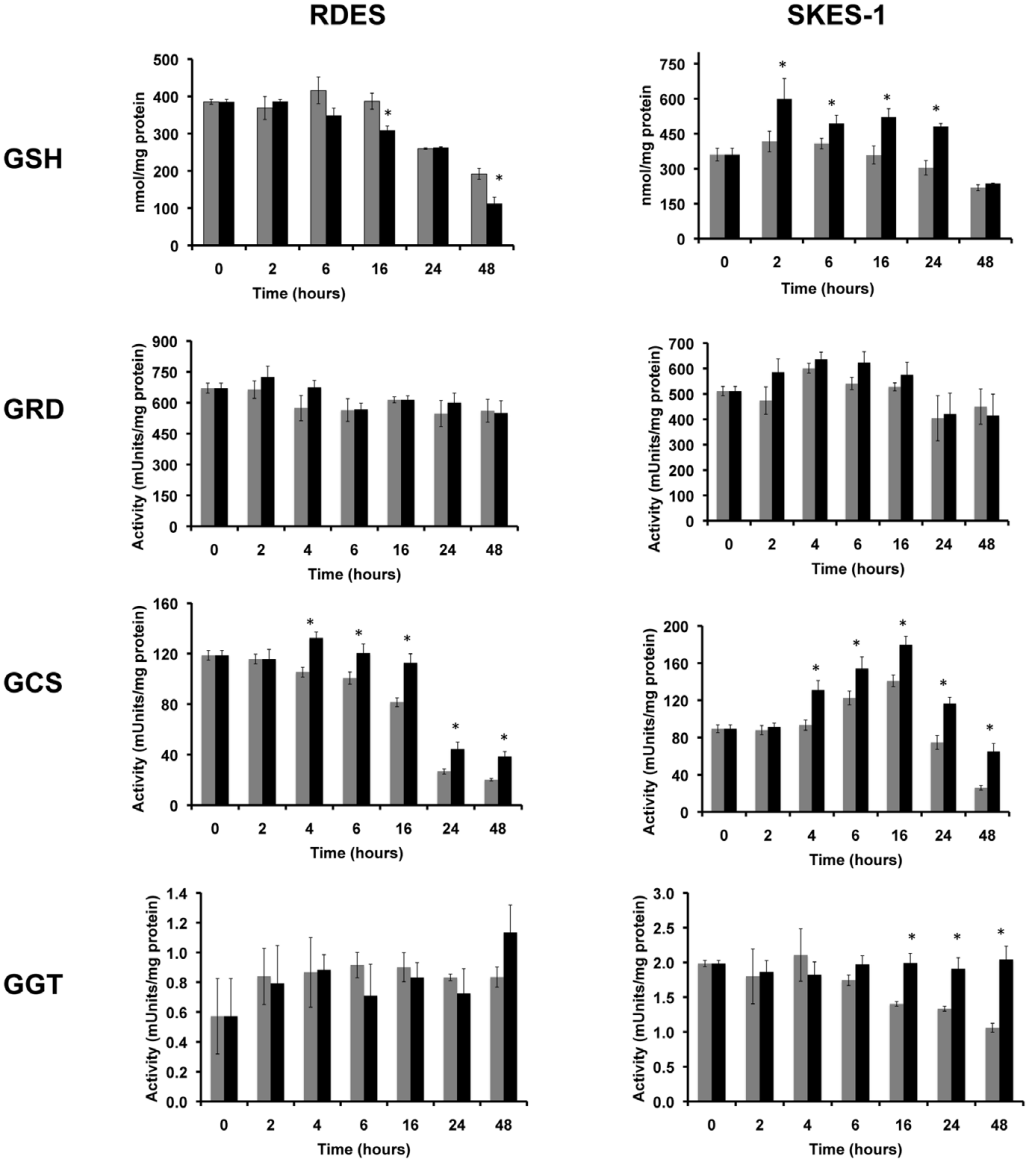
GSH levels and the activity of GSH-regulatory enzymes in RDES and SKES-1 cells. Cells were harvested at the time points indicated for determination of GSH levels and the activities of GRD, GCS, and GGT1 as described in the text. Each bar represents the mean ± SEM of n = 6 determinations. Grey bars = normoxia; Black bars = hypoxia. *Hypoxia values significantly different from those in normoxia at p≤0.01.

The effect of hypoxia on GSH levels in ESFT cells was cell-line-specific (Figure 3). In RDES cells GSH levels decreased with time between 0 and 48 hours in both normoxia and hypoxia; however GSH levels were significantly decreased in hypoxia compared to levels in normoxia at 16 and 48 hours (Figure 3, p<0.01). In SKES-1 cells, GSH levels remained constant over 48 hours in normoxia but were significantly increased in hypoxia compared to levels in normoxia between 2 and 24 hours (Figure 3, p<0.001).

#### Glutathione Reductase (GRD)

Hypoxia had no significant effect on GRD activity in RDES or SKES-1 cells (Figure 3), possibly suggesting that the capacity of ESFT cells to regenerate GSH was not affected by hypoxia.

#### γ-Glutamylcysteine synthetase (GCS)

GCS enzymatic activity decreased with time in both RDES and SKES-1 cells maintained in normoxia and hypoxia (Figure 3). However, for both cell lines GCS activity was significantly higher in hypoxia compared to normoxia at all time points from 4–48 hours (p<0.001). The higher GCS activity in hypoxia is consistent with the hypothesis that hypoxia increases the synthesis of GSH which potentially contributes to the decreased sensitivity of ESFT cells to ROS-inducing agents in this environment.

#### γ-Glutamyl transferase (GGT)

The enzymatic activity of GGT remained constant at all time points for both RDES and SKES-1 cells but was significantly decreased in normoxia for SKES-1 cells from 16 to 48 hours (Figure 3, p<0.001). This suggests that in some cell types breakdown of GSH may be reduced in hypoxia, and could contribute to the decreased sensitivity to some chemotherapeutics in such environments.

### GSH levels are linearly correlated with enzymatic activity of its regulatory enzymes

**Figure 4:**
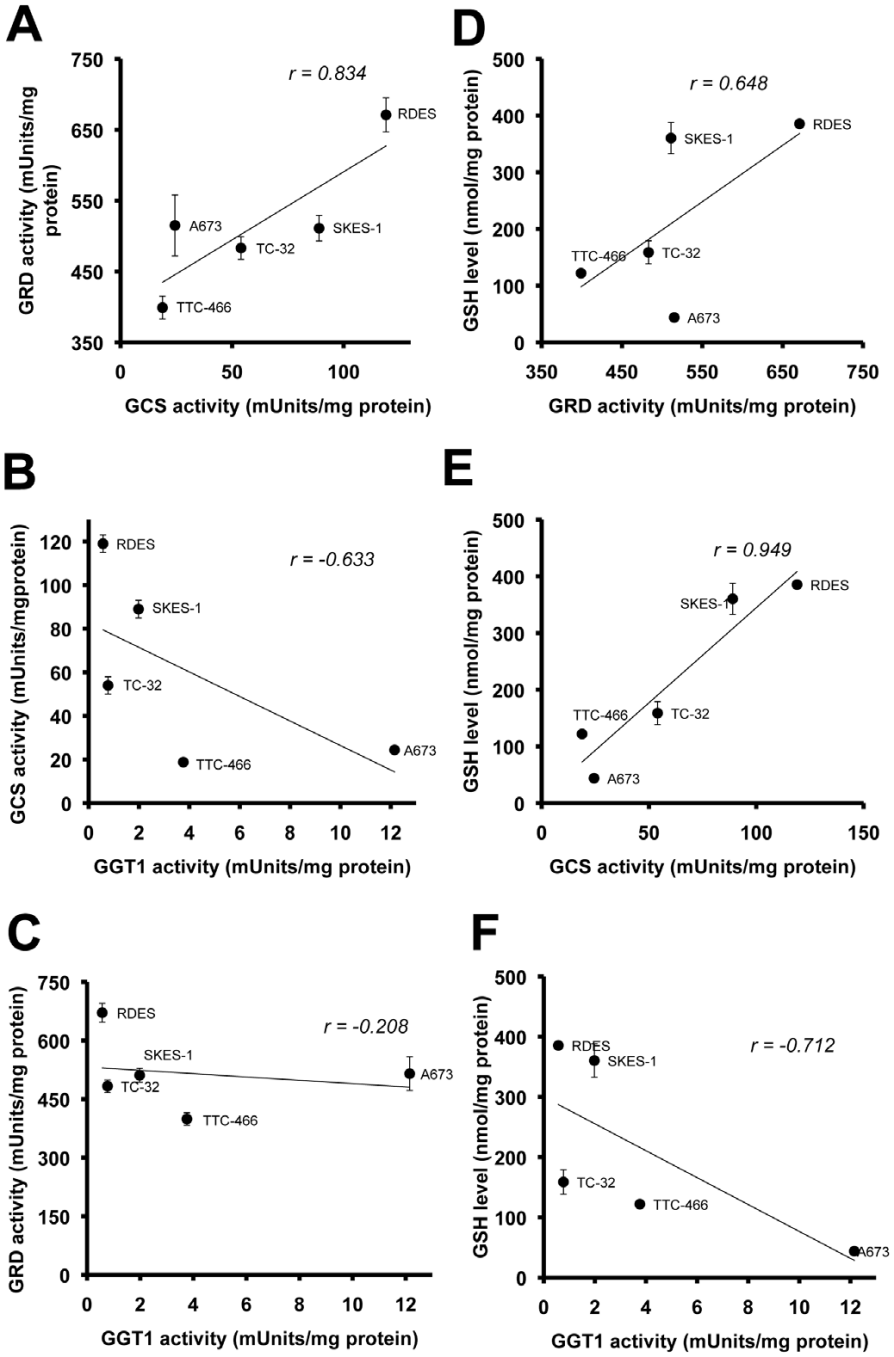
Pearson correlation modelling of the relationships between GSH and its regulatory enzyme activities in ESFT. The parameters were measured in ESFT cells cultured under normoxic conditions in the absence of fenretinide. (A–C) Relationships between the enzymatic activities of GSH regulatory enzymes. (D–F) Correlation between activities of GSH-regulatory enzymes and GSH levels in ESFT cells.

In order to establish the wider putative role of GSH in ESFT cells we investigated the relationship between the activity of GSH regulatory enzymes and cellular GSH levels in the absence of chemotherapeutic drugs in normoxia across a panel of 5 ESFT cell lines. Pearson correlation was used to test the statistical significance of these relationships. A strong positive correlation between GRD and GCS activity (Figure 4A, ***r*** = 0.834) and between the activities of these two enzymes and levels of GSH (Figure 4D–E, ***r*** = 0.648 and ***r*** = 0.949, respectively) was observed. Weak to moderate negative correlations between GGT activity and GCS activity (Figure 4B: ***r*** = −0. 633), GRD activity (Figure 4C: ***r*** = −0. 208), and GSH levels (Figure 4F: ***r*** = −0.712) in ESFT cells were also observed. These results suggest that biosynthesis and regeneration might be the two reactions that are most critical for maintenance of intracellular GSH levels in ESFT.

### Modulation of intracellular GSH levels and the response of ESFT cells to fenretinide

Whether GSH plays a role in modulating the sensitivity of ESFT cells to fenretinide in normoxia and hypoxia was investigated by (i) depleting intracellular GSH levels using targeted siRNA-mediated knockdown of GCS and GRD, and (ii) increasing intracellular levels of GSH by incubating cells with *N*-acetylcysteine (NAC), and then testing the sensitivity of these cells to fenretinide when maintained under normoxia or hypoxia.

#### (i) Effect of siRNA-dependent decrease in GSH levels on response of RDES and SKES-1 cells to fenretinide in normoxia and hypoxia

**Figure 5:**
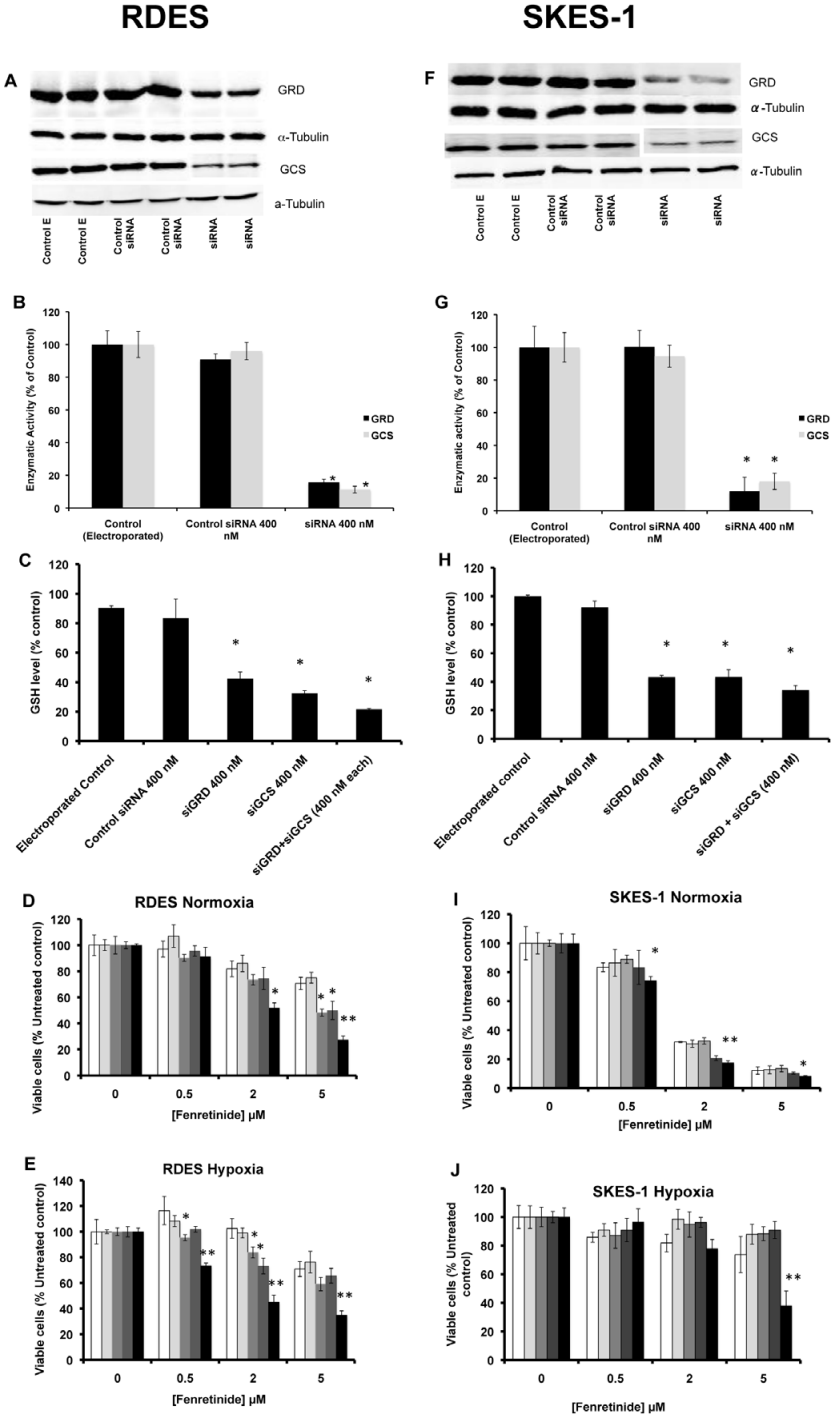
Effects of siRNA-targeted knockdown of GRD and GCS on sensitivity of RDES (A–E) and SKES-1 (F–J) cells to fenretinide. Western blots (Panels A & F) show decreased GRD and GCS protein levels in RDES and SKES-1 cells electroporated with siRNA. Enzymatic activities of GRD and GCS were decreased in both RDES (Panel B) and SKES-1 (Panel G) cells following electroporation with siRNA, *p<0.0001. GSH levels were also significantly decreased in RDES (Panel C) and SKES-1 (Panel H) cells following electroporation with siRNA, *p<0.0001. Sensitivity of ESFT cells to fenretinide was improved by decreasing GSH levels in both RDES (Panels D & E) and SKES-1 (Panels I & J) cells in normoxia and hypoxia. For panels D,E,I, and J, white bars = electroporated control; light grey bars = control non-specific siRNA; grey bars = siGRD; dark grey bars = siGCS; Black bars = siGRD+siGCS (400 nM each), *p≤0.01; **p≤0.0001.

Having identified GRD and GCS as the two enzymes that are important for regulating intracellular GSH levels in ESFT, we investigated whether siRNA-mediated knockdown of these two enzymes might sensitize ESFT cells to fenretinide. The magnitude of siRNA-mediated knockdown of GRD and GCS catalytic subunit on protein levels, enzymatic activity, and GSH levels are shown in Figure 5A, B, & C for RDES and Figure 5F, G, H, & J for SKES-1 cells. Treating siRNA-loaded RDES cells with fenretinide significantly increased the sensitivity of these cells to fenretinide in both normoxia and hypoxia (Figures 5D & 5E, p<0.001); the combined knockdown of GRD and GCS catalytic subunit using siRNA achieved a greater effect than either siRNA alone (p<0.0001). In hypoxia, siRNA-mediated knockdown of either GRD or GCS alone failed to sensitize SKES-1 cells to fenretinide (Figure 5J), however double knockdown of the two enzymes together enhanced fenretinide cytotoxicity (Figure 5J, p<0.0001). These results are consistent with the regulatory role of these enzymes on GSH levels establish earlier, and suggest that targeting multiple GSH regulatory enzymes may be needed to overcome hypoxia-induced GSH-dependent drug resistance of some ESFT cells. The results of siRNA-mediated knockdown of the GCS catalytic subunit in the two ESFT cell lines are thus consistent with our previous observations that showed increased sensitivity to fenretinide following inhibition of GCS enzymatic activity by BSO [25].

#### (ii) Effect of NAC-dependent increase in GSH levels on response of RDES and SKES-1 cells to fenretinide in normoxia and hypoxia

**Figure 6:**
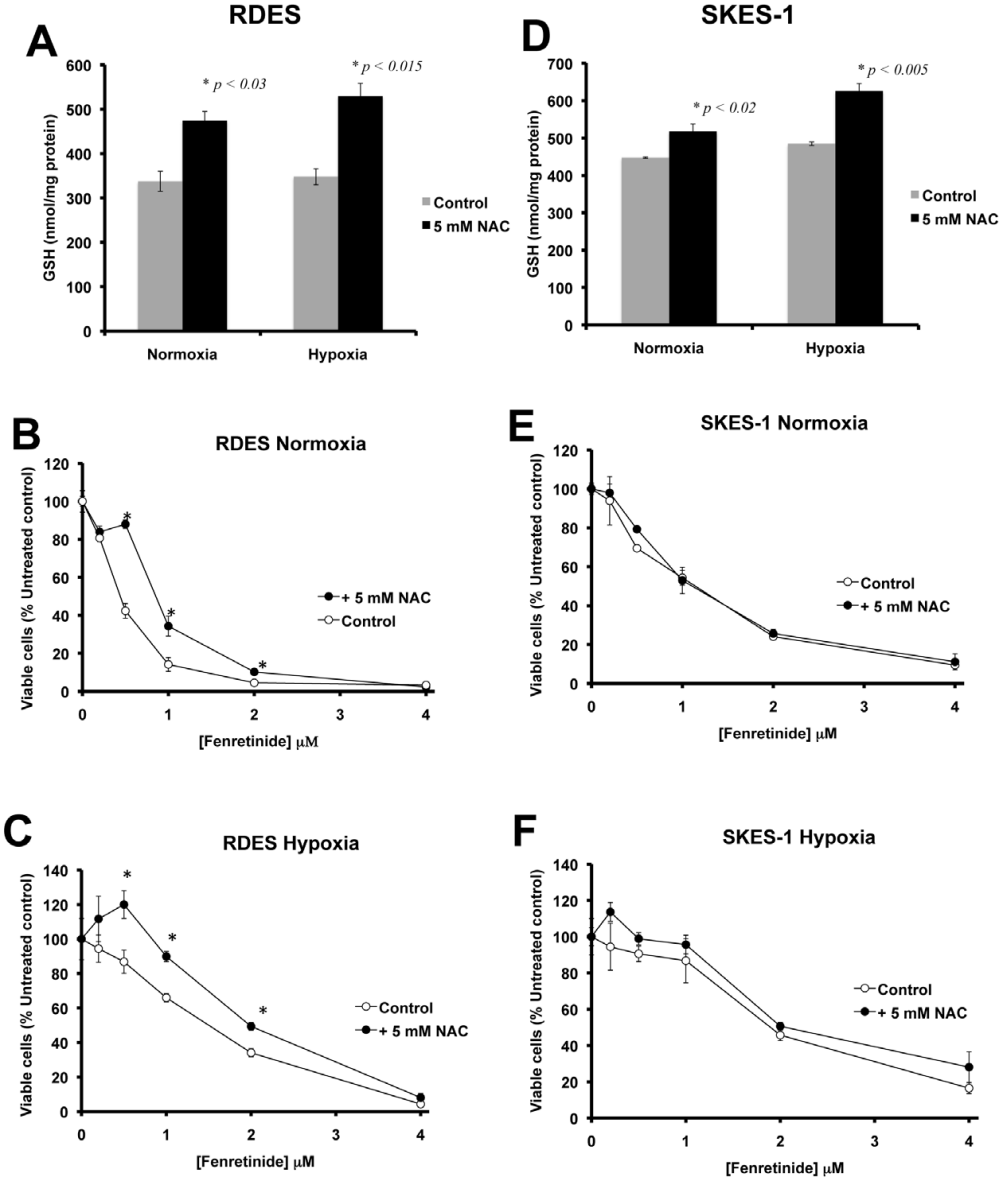
The effect of increasing GSH levels by NAC supplementation on sensitivity of RDES (Panels A–C) and SKES-1 (Panels D–F) cells to fenretinide. NAC supplementation significantly increased GSH levels in RDES (Panel A) and SKES-1 (Panel D) by between 10 and 50% compared to un-supplemented controls. The effects of increasing GSH levels in RDES (Panels B–C) and SKES-1 (Panels E–F) cells were cell line-specific. *Number of viable cells was significantly different between normoxia and hypoxia, specific IC-50 values are reported in the text.

We hypothesized that if GSH was a critical factor in reducing the sensitivity of ESFT to fenretinide in hypoxia, then an increase in intracellular GSH levels should augment this effect. To test this hypothesis we used NAC to increase intracellular GSH levels in ESFT cell lines and then tested their sensitivity to fenretinide in both normoxia and hypoxia. Treatment of RDES and SKES-1 cells with 5 mM NAC for 24 hours significantly increased cellular levels of GSH (Figure 6A and D). Increasing cellular GSH levels in RDES cells caused the cells to become more resistant to fenretinide-induced cell death in normoxia (Figure 6B: IC-50 value NAC treated = 1.41 µM compared to IC-50 value control = 0.85 µM, p<0.0001) and augmented the resistance to fenretinide in hypoxia (Figure 6C: IC-50 value NAC treated = 2.00 µM compared to IC-50 control = 1.86 µM, p<0.0091). In SKES-1 cells, the result of increasing GSH levels on sensitivity to fenretinide was less dramatic in both normoxia (Figure 6E: IC-50 value NAC treated = 1.77 µM compared to IC-50 value control = 1.66 µM, p<0.07) and hypoxia (Figure 6F: IC-50 value NAC treated = 2.42 µM compared to IC-50 value control = 2.31 µM, p<0.36) compared to changes observed in RDES cells. The augmentation of RDES resistance to fenretinide in hypoxia after artificially increasing GSH levels supports the hypothesis that GSH plays a role in the reduced sensitivity of ESFT cells to fenretinide in this microenvironment. However, the lack of effect of increasing GSH on sensitivity of SKES-1 cells to fenretinide in normoxia or hypoxia demonstrates that the involvement of GSH in modulating sensitivity of ESFT to fenretinide is heterogeneous, and strongly suggests the involvement of other factors in hypoxia-induced drug resistance.

## Discussion

Identifying factors that contribute to the reduced sensitivity of ESFT to therapeutics in low oxygen environments could enable the design of novel therapies that are more effective in treating disease. In this study we identified GSH as one factor that may be important in modulating the response of some ESFT cells to fenretinide in hypoxia. However the response of the GSH antioxidant system in the 5 ESFT cell lines to hypoxia was cell-line-specific and the response of ESFT cell lines to modulation of intracellular GSH levels was heterogeneous. This is consistent with our view that the GSH antioxidant system may play a role in hypoxia-induced drug resistance in some but not all ESFT, and the proposition that this should be characterised in tumours to establish which patients might benefit from therapies exploiting regulators of the GSH antioxidant defence system. Since GSH is an alternative cellular target to most conventional chemotherapeutics, we have taken the view that future combination treatments exploiting the GSH antioxidant defence system may potentially improve treatment outcome in selected patients with ESFT [7].

The regulation of cellular GSH occurs at multiple levels involving biosynthesis, regeneration, and degradation. Results of this study show a strong positive correlation between GSH levels and GCS activity, suggesting that *de novo* synthesis may be more important for GSH preservation in ESFT than recycling or degradation. The GCS protein is a heterodimer composed of a large catalytic subunit of molecular weight 78 kDa and a smaller regulatory subunit of 31 kDa. Transcriptional regulation of both GCS subunits is mediated by the nuclear factor (erythroid-derived 2)-like 2 (Nrf-2) transcription factor, the master switch for cellular antioxidant defence [31], whereas post-transcriptionally the activity of the holoenzyme is affected by phosphorylation [32]. Inhibition of GCS enzyme leads to GSH depletion within cells [4], [13] whereas cellular stresses that produce ROS can increase GSH levels via induction of GCS [32], [33]. There is currently no information on how GCS activity (hence cellular GSH levels) is affected or regulated by hypoxia *in vitro*. Here we report for the first time that GCS enzymatic activity was increased by hypoxia in two ESFT cell lines. On the basis of these results we postulate that the higher GCS activity in hypoxia may indirectly contribute to reduce sensitivity of ESFT cells to fenretinide by increasing the GSH antioxidant defence against drug-induced oxidative stress or dampening of pro-apoptotic stimuli [3]. Therefore in some tumours targeting GCS may provide an opportunity to increase sensitivity to fenretinide under hypoxic conditions.

The results in this manuscript on the effects of hypoxia on ROS production and the sensitivity of ESFT cells to chemotherapeutic agents are at variance with some of the current literature. In this study we observed that the level and time course of ROS increase following treatment of ESFT cells with fenretinide were similar in hypoxia and normoxia, suggesting the differential response of ESFT cells to fenretinide in hypoxia and normoxia was not a result of differences in the levels of ROS produced. This is in contrast to results reported by Batra *et al*. [10] who described a decrease in ROS levels in hypoxia compared to normoxia 6 hours after treating SK-N-MC cells with fenretinide [10]. We have not investigated the SK-N-MC cell line and so cannot directly compare our data with that of Batra *et al.* [10], however this discrepancy may reflect differences in methodology and/or timing of ROS assays. Our own results are consistent with previous publications demonstrating that ROS production is an early initiating event in fenretinide-induced death in ESFT [24]. These discrepancies may also suggest that fenretinide induces cell death through other non-ROS mechanisms such as ceramide production [10] or through death receptors [34] that could be cell line-specific.

In this study we have found a significant reduction in the sensitivity of all ESFT cells to fenretinide in hypoxia, but the resistance to doxorubicin and vincristine was marginal and cell-line-specific. This is in contrast to the findings of Kilic and co-workers who reported decreased sensitivity of the A673 cell line to doxorubicin and vincristine in hypoxia [12]. Two possible explanations for this discrepancy could be differences in culture conditions between the two studies and/or the methods used to assess and analyze cell death in drug-treated cells. In this study ESFT cells were maintained in a hypoxic environment (1% O_2_, 5% CO_2_, and 94% N_2_), whereas in the study from Kilic *et al*. [12] experimental conditions were anoxic (5% CO_2_ and 95% N_2_), hence the response of cell lines to drug treatment in these two environments may differ. Furthermore, different methods to assess the efficacy of the drugs were used. Kilic *et al*. [12] used a FACS-based method to measure DNA fragmentation of PI-stained cell nuclei [12] whereas we used the trypan blue exclusion assay to measure viable cell number and the FACS-based Annexin V-PI to quantify the level of apoptotic cell death. While these three methods are all useful for assessing cell death in a range of cell lines and conditions, the assay endpoints are different so consequently results from one assay may not be directly comparable to those of another within a given time and experimental design. We have therefore taken the view that the results obtained with one method should be validated by at least a second methodological approach and have utilised the trypan blue exclusion assay and Annexin V-PI to quantify effects in this study.

The EWS/Fli1 transcription factor plays a major role in regulating apoptosis in ESFT both *in vitro* and *in vivo* [35], [36] and enhances the drug resistant phenotype of ESFT cells in hypoxia in a HIF-1α-dependent manner [37]. Hence to eliminate the influence of different EWS/Fli1 fusion types on sensitivity to fenretinide toxicity in this study we used RDES and SKES-1 cell lines that are both EWS/Fli1 fusion type II and hemizygous null for p53 to study the role of GSH in greater detail. Using these two ESFT cell lines with some common genetic features we observed that the role of GSH in mediating the resistance to fenretinide was cell-line-specific. This implicates the induction of other drug resistance mechanisms in the response of ESFT to fenretinide. These are most likely HIF-1α regulated and may involve decreased senescence [38], induction of an autophagy-dependent pro-survival mechanism [39], [40], or hypoxia-induced modulation of factors involved in the initiation of cell death such as ceramide [10].

In conclusion, this study demonstrates that the role of GSH in modulating the sensitivity of ESFT cells to fenretinide is heterogeneous depending on the environment and cell type. While manipulation of GSH levels using siRNA-targeted knockdown of its regulatory enzymes or artificially increasing its intracellular levels using NAC supplementation partly supports the hypothesis that GSH could be involved in hypoxia-induced drug resistance, a major caveat is that its role appears to be cell line-specific. This is likely to limit the value of targeting GSH alone as a therapeutic strategy for overcoming hypoxia-induced drug resistance of ESFT.

## Materials and Methods

### Chemicals and reagents

Cell culture media: RPMI 1640, DMEM, MEM, and McCoy's were purchased from Sigma -Aldrich (UK). Opti-MEM® I reduced-serum media was procured from Invitrogen (Paisley, UK). The chemotherapeutic drugs fenretinide, doxorubicin, and vincristine were also purchased from Sigma-Aldrich (UK) and dissolved in absolute ethanol (fenretinide) or water. Bovine foetal calf serum (FCS) was obtained from Seralab (Sussex, United Kingdom) and stored frozen in single use aliquots at −20°C. The redox-sensitive dye used for ROS detection, 5-(and 6-)-chloromethyl-2′-7′-dichlorofluorescein diacetate acetyl ester (CM-H_2_DCFDA) and the Alexa Fluor® 680 secondary antibodies were purchased from Molecular Probes (Eugene, OR, USA). The Annexin V-FITC Apoptosis Detection kit for assessing cell death was purchased from BD Biosciences (UK). The glutathione (GSH) standards, adenosine-5′-triphosphate (ATP), 2,3-naphthalenedicarboxaldehyde (NDA), 5-sulfosalicylic acid (SSA), γ-glutamyl-*p*-nitroanilide (GPNA), *p*-nitroaniline, L-glutamic acid, L-cysteine, N-acetylcysteine, L-serine, L-buthionine-S,R-sulfoximine (BSO), metaphosphoric acid, and the CellVue®Claret kit used for measuring cell proliferation were procured from Sigma-Aldrich (UK). The antibodies for GRD (ab16801), GCS (ab40929), and GLUT1 (ab32551) were purchased from Abcam® (Cambridge, UK). The GRD activity kit was purchased from Cayman Europe (Estonia). The pre-designed *Silencer*® Select siRNAs used for the knockdown of GCS (s5800) and GRD (s6249), and the scrambled siRNA used to control for the non-specific effects of siRNA (negative control) were purchased from Applied Biosystems (UK).

### Cell culture

Five substrate-adherent ESFT cell lines A673, RDES, SKES-1, TC-32, and TTC-466 were used in this study. The A673, RDES, and SKES-1 cells were purchased from the American Type Culture Collection, Manassas, VA. TC-32 and TTC-466 cell lines were kind gifts from Dr J. Toretsky (Division of Pediatrics, University of Maryland, Baltimore, MD) and Dr P. Sorenson (British Columbia Children's Hospital, Vancouver, British Columbia, Canada), respectively. Cell line identity was confirmed by cell morphology checks and bimonthly growth curve analysis before and during the course of the study as per ATCC and European LGC recommendations. In addition, checks on cell line-specific characteristicss that include G-banding for chromosome number, RT-qPCR to confirm fusion transcripts, and multiplex ligation-dependent probe amplification (MLPA) analysis to verify p16 status of each cell line were done every 6–12 months. All cell lines were cultured without antibiotics in medium containing 10% FCS except where indicated. Mycoplasma tests (MycoAlert® Mycoplasma detection assay, Lonza) were carried out bi-monthly during the course of the study to ensure cells were free from contamination in the absence of antibiotics. A673 cells were grown in DMEM medium, whereas RDES, TC-32, and TTC-466 cells were grown in RPMI 1640 media; the media for TTC-466 cells was supplemented with 10% sterile conditioned media. SKES-1 cells were grown in McCoy's medium containing 15% FCS. All incubations and cell treatments were carried out in humidified normoxic incubators (Sanyo Gallenkamp, Loughborough, UK) containing 5% CO_2_ and 95% air at 37°C, or hypoxic incubators containing 1% oxygen, 5% CO_2_ and 94% nitrogen at 37°C.

### Effect of chemotherapeutic drugs on ESFT cell viability

ESFT cells (5×10^4^ cells/well) were seeded into 24-well Primaria plates and allowed to adhere overnight in a normoxic incubator. Cells were then incubated in normoxia or hypoxia and treated with the following chemotherapeutic drugs: fenretinide (0–15 µM), doxorubicin (0–50 nM), and vincristine (0–20 nM). Cell viability was assessed by the trypan blue exclusion assay after 48 hours. These three drugs were selected based on their distinct mechanisms of action for inducing cell death. Fenretinide induces cell death in malignant cells by increasing levels of intracellular ROS to activate the intrinsic mitochondrial death cascade [41] whereas doxorubicin and vincristine do so by DNA intercalation or inhibition of DNA topoisomerase II [42], [43], and inhibition of mitosis by disrupting microtubule formation [44], respectively.

### ESFT cell proliferation and apoptosis

Whether hypoxia affected proliferation or apoptosis of ESFT cells was evaluated using the CellVue® Claret and annexin-V/PI assays, respectively.

To measure proliferation of cells in normoxia and hypoxia, the CellVue®Claret assay was utilised according to manufacturer's instructions. Briefly, cells were harvested by trypsinization, washed three times with serum-free media and then resuspended in staining media (company proprietary) at a density of 2×10^7^ cells/ml. An equal volume of 4 µM CellVue® Claret dye (CVC) in staining media was then added to the cells to give a final concentration of 2 µM CVC. After mixing, the cells were incubated for 5 minutes at room temperature and then the reaction was stopped by addition of an equal volume of serum. The cells were then washed three times in serum-containing media, viable cell number counted, and cells seeded into 6-well plates at a density of 2×10^5^ cells/well before placing in either hypoxic or normoxic incubators. After 24, 48, and 72 hours the cells were harvested by trypsinization for measurement of fluorescence by FACS analysis (excitation wavelength 655 nm; emission wavelength 675 nm). The proliferation of cells is directly proportional to the decrease in mean fluorescence of the CVC dye with time in live cells.

The effects of chemotherapeutic drugs on cell proliferation were assessed in similarly designed experiments but the chemotherapeutic drugs were added 24 hours after the CVC dye and the fluorescence measured subsequently at 24 and 48 hours. The level of apoptosis was assessed by staining cells with Annexin V and PI according to the protocol described in the Annexin V-FITC Apoptosis Detection kit from BD Biosciences (UK); data acquisition and analysis was performed using FACS and CellquestPro™ software.

### Measurement of intracellular ROS levels

The fluorescent dye CM-H_2_DCFDA was used to assess the levels of ROS in ESFT cells by FACS analysis as previously described [25]. For ROS measurements in hypoxic cells, cells were taken out of the incubator for harvesting under normoxic conditions and quickly replaced in incubator after labelling with CM-H_2_DCFDA dye. After the 15 min incubation period cells were twice washed with PBS by spinning at 400× g at room temperature and FACS analysis of the dye-labelled cells was performed as quickly as possible within 30 minutes after harvesting.

### Western blotting

GRD, GCS, and GLUT1 protein levels were assessed using the Western blotting method previously described [25]. Primary antibodies were diluted as recommended by supplier and the secondary antibodies were diluted 1∶5000. Protein bands were detected using the Odyssey™ Infrared detection system (Li-COR Biosciences, Lincoln, NE).

### Measurement of GSH levels

ESFT cells (2×10^5^) were placed into 10 cm^2^ dishes and allowed to adhere overnight under normoxic conditions. Half the dishes were then placed in a hypoxic incubator and the rest maintained in normoxia. Cells were harvested after 0, 2, 4, 6, 16, 24, and 48 hours by scraping into ice-cold PBS and centrifugation at 400× g for 5 minutes, and PBS removed by aspiration. The cell pellets were immediately homogenized in 500 µl MES buffer and GSH levels were determined in fresh cell extracts using the enzymatic recycling method as previously described [25]. The GSH level was normalized to total cellular protein concentration estimated in cell extracts using the Bio-Rad DC Protein Assay kit (Bio-Rad, Herts. UK).

### Measurement of GSH regulatory enzyme activities

These experiments were set-up was as described above using cells that were sub-confluent at the time of harvesting. Cells were homogenized in 50 mM potassium phosphate (pH 7.5) containing 1 mM EDTA for the GRD assay; 120 mM Tris-glycyglycine buffer (120 mM Tris, 80 mM glycylglycine, 1% Triton X, 10% glycerol, pH 8.0) for the GGT assay; and TES/SB buffer (20 mM Tris, 1 mM EDTA, 250 mM sucrose, 20 mM sodium borate, 2 mM serine) for the GCS assay. Homogenates were centrifuged at 12 000× g (15 minutes, 4°C) and the supernatants maintained on ice for determination of enzyme activities. The protein concentration of cell supernatants was measured using the Bio-Rad DC Protein Assay kit (Bio-Rad, Herts. UK) and enzyme activities reported as mUnits/mg of protein, where a unit of activity is the amount of enzyme required to convert one μmole of substrate to product per minute at 25°C.

The Glutathione Reductase Assay Kit ™ (Cayman Europe, Estonia) was used to measure GRD activity in ESFT cell extracts by determining the rate of NADPH oxidation according to manufacturer's instructions. GGT activity was measured according to the kinetic procedure [45]; in this method the substrate analogue GPNA is cleaved by GGT present in cell extracts to form *p*-nitroaniline, production of which is determined spectrophotometrically at 405 nm. This GCS assay is an adaptation of the method previously described [46], in which GCS in cell extracts synthesizes γ-glutamylcysteine which is then reacted with NDA to form a highly-fluorescent product that can be measured fluorimetrically at 520 nm [46].

### Electroporation of cells with GRD and GCS siRNA

ESFT cells were electroporated with siRNA targeting either GRD (siGRD) or GCS (siGCS) to achieve knockdown of these proteins. The siGCS (s5800) targets the catalytic subunit of the GCS enzyme for knockdown. Briefly, (2–10)×10^5^ cells were suspended in 0.4 ml of culture media in 0.4-cm cuvettes (Bio-Rad). After addition of siRNA to the cuvette, cells were subjected to an electrical pulse (Voltage = 0.4 kV, Capacitance = 500 µF) for 12–15 ms in a Gene Pulser II instrument (Bio-Rad, UK). Two control groups consisted of cells electroporated with scrambled non-specific (negative control) siRNA (Applied Biosystems, UK) and cells electroporated in the absence of siRNA. After electroporation cells were seeded into six well plates in normoxia and cells harvested at 24–72 hours for the preparation of protein extracts and western blotting (see above) to evaluate the magnitude and duration of GRD and GCS knockdown. The effect of fenretinide (0.5–5.0 µM) on viable cell number of cells maintained in the hypoxic incubator and under normoxia for 48 hours was then compared; viable cell number was assessed using the typan blue exlusion assay.

### Artificially increasing cellular GSH levels in ESFT cells

Cellular levels of GSH were artificially increased by treating ESFT cells (2×10^5^) with a non-toxic concentration of NAC (5 mM) for 24 hours in 6-well primaria plates. NAC supplementation increases intracellular GSH levels by providing the essential amino acid cysteine that is required for GSH biosynthesis [47]. Control cells were treated with vehicle only. After 24-hour, cells from one plate in each group were harvested as described above to determine the baseline level of GSH. Media was then aspirated from the remaining plates and replaced with fresh media containing fenretinide (0.5–4.0 µM). The fenretinide-treated cells were then incubated in normoxia or hypoxia for 48 hours after which viable cell number was analyzed by the trypan blue exclusion assay.

### Statistical analyses

All data were analyzed using GraphPad Prism® statistical software (GraphPad Software Inc., La Jolla, CA). Only *p* values less than 0.05 (p<0.05) were considered statistically significant.

Differences in viable cell number or proliferation of normoxia- and hypoxia-cultured ESFT cells were tested by linear regression analysis of the slopes of log-linear plots of the logarithm of viable cell number or CVC fluorescence (proliferation) against time. Results from the Annexin V-PI assays to assess cell death after treatment with chemotherapeutic agents in normoxia and hypoxia were compared statistically using the non-parametric Mann-Whitney-Wilcoxon rank sum test. GSH levels and activities of GRD, GCS, and GGT enzymes between cells grown in normoxia and hypoxia at each time point were compared statistically using 2-way ANOVA followed by Bonferroni post-hoc tests. The correlations between GRD, GCS, GGT and GSH levels were examined using Pearson's correlation.

## Supporting Information

Supporting Information S1 (PDF)
