# Supplementary material for: Heterogeneous Role of the Glutathione Antioxidant System in Modulating the Response of ESFT to Fenretinide in Normoxia and Hypoxia
Source: PLoS One. 2011 Dec 8;6(12):e28558. doi: 10.1371/journal.pone.0028558 (PMC3234283; doi:10.1371/journal.pone.0028558)
Supplement: Supporting Information S1 — (PDF) [file pone.0028558.s001.pdf]

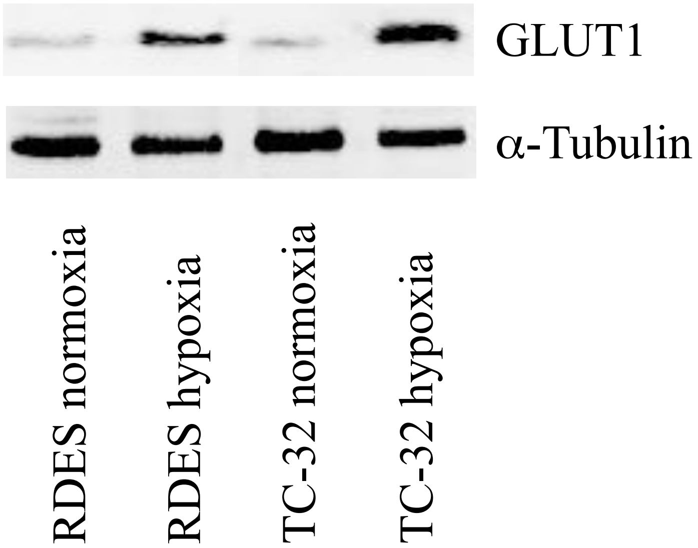

Figure S1: Stabilization of the glucose transporter GLUT1 expression in RDES and TC-32 cells by hypoxia over 48 hours. ESFT cells were placed in 10 cm^2 dishes at a density of 2 x 10^5 cells/dish and allowed to adhere to plastic overnight in normoxia after which half the dishes were placed in a hypoxic incubator and the rest maintained in normoxia. After 48 hours the cells were harvested by scraping into RIPA buffer containing protease inhibitors on ice (see main text) and 50 μg protein extracts were separated by SDS PAGE. Primary antibodies for GLUT1 (ab32551) were purchased from Abcam (Cambridge, UK) and were used at a concentration of 1 μg/ml overnight at 4 ^oC. Secondary antibodies (Alexa Fluor® 680) were purchased from Molecular Pribes (Eugene, USA). Protein bands were detected using the Odyssey™ Infrared detection system (see main text for details).

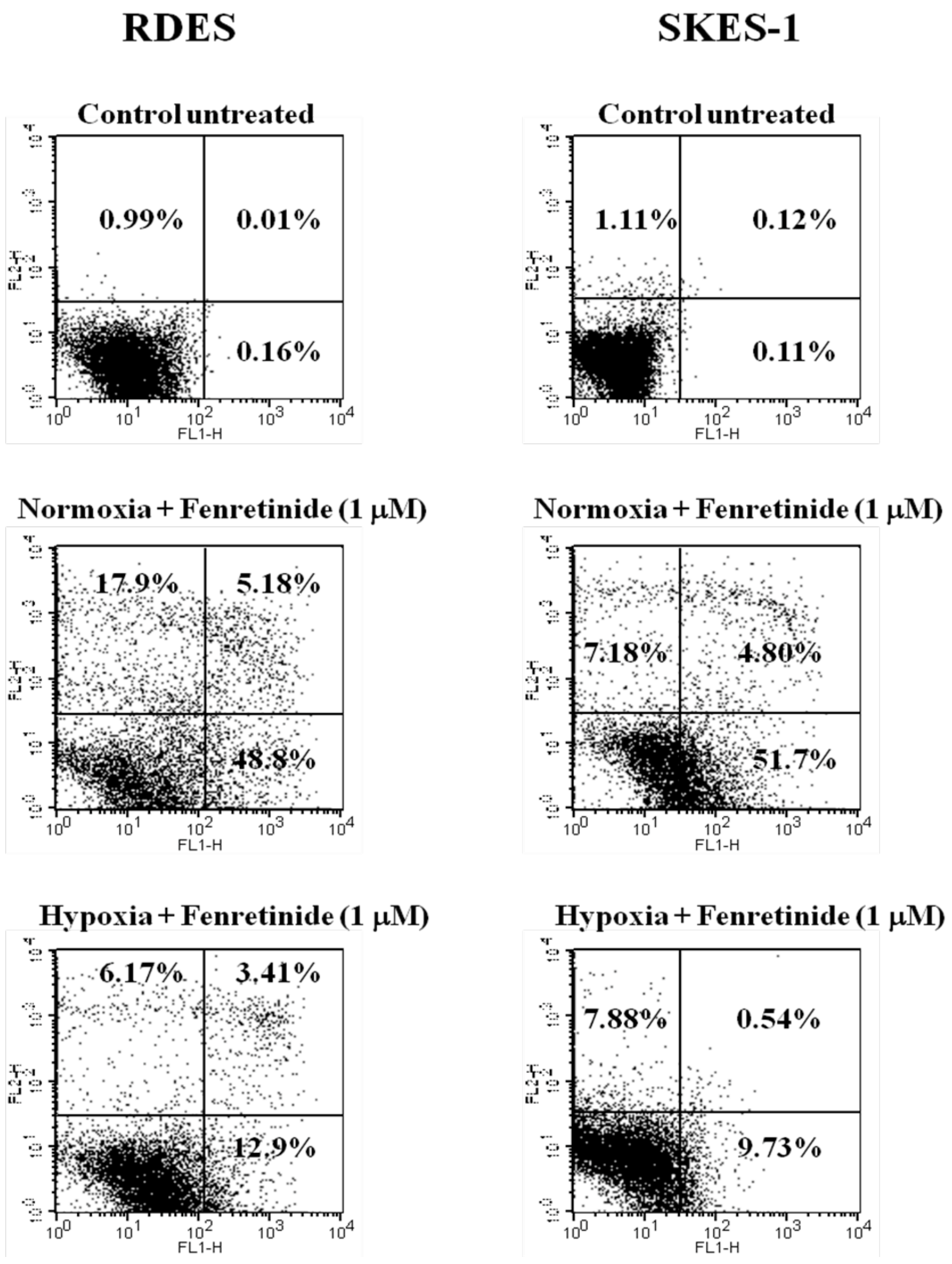

Figure S2: Representative graphs for the cell death assays assessed by Annexin V and PI staining of fenretinide-treated ESFT cells. Cells were incubated with fenretinide in normoxia or hypoxia as described in the text and the level of apoptosis was assessed by staining with Annexin V and PI according to the protocol described in the Annexin V-FITC Apoptosis Detection kit from BD Biosciences (UK); data acquisition and analyses were performed using FACS and CellquestPro™ software.

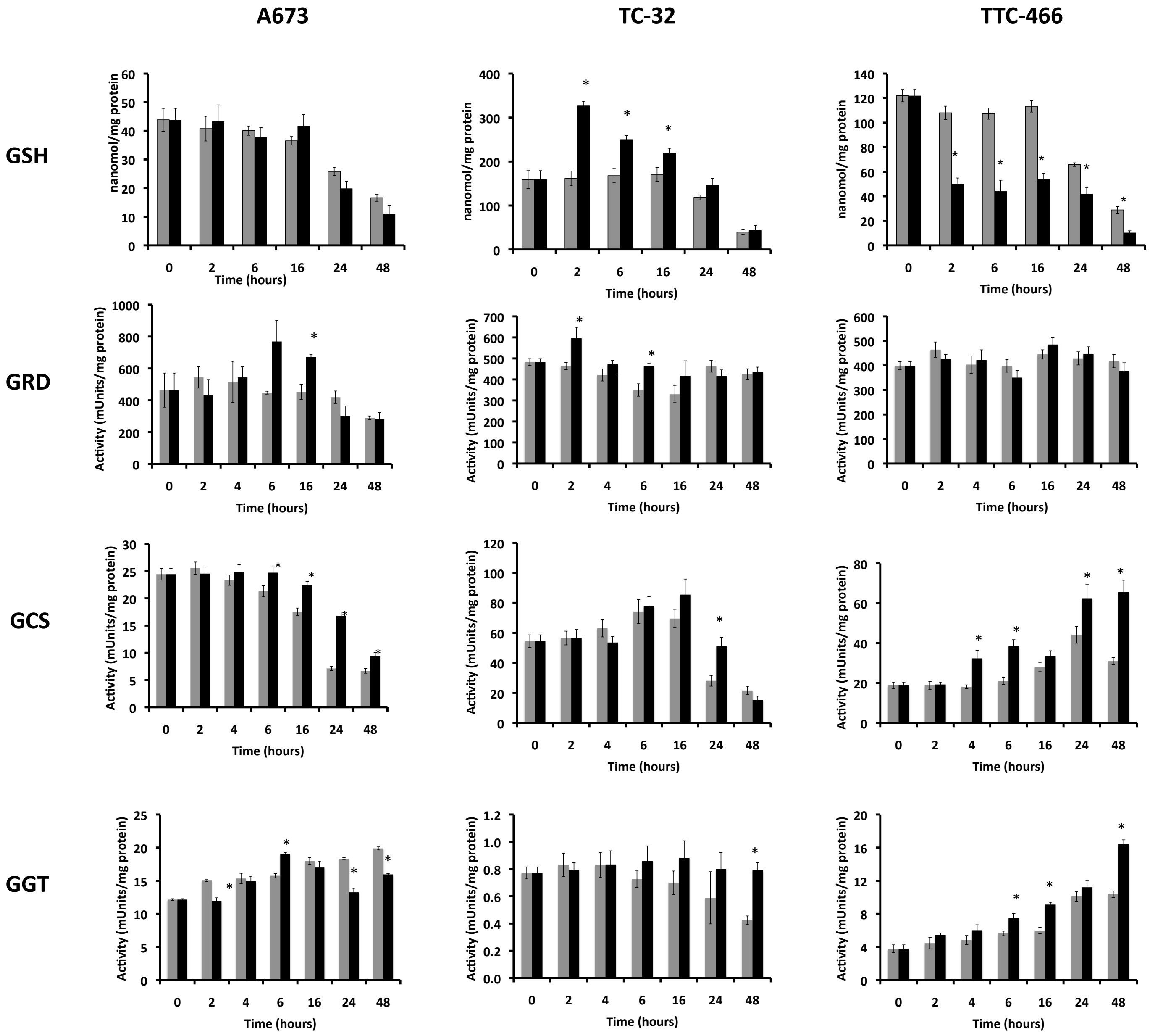

Figure S3: Changes in GSH levels and activities of GSH-regulatory enzymes of A673, TC-32 and TTC-466 cells cultured in normoxia and hypoxia over 48 hours. ESFT cells were placed in 10 cm^2 dishes at a density of 2 x 10^5 cells/dish and allowed to adhere to plastic overnight in normoxia after which half the dishes were placed in a hypoxic incubator and the rest maintained in normoxia. Cells were harvested at the time points indicated for determination of GSH levels and the activities of GRD, GCS, and GGT by methods described in the text. Each bar represents the mean ± SEM of n = 6 determinations. Grey bars = normoxia; Black bars = hypoxia. *Hypoxia values significantly different from normoxia at maximum p value ≤ 0.01.

Table S1: Summary of the effects of doxorubicin and vincristine on proliferation of ESFT cells in normoxia and hypoxia over 48 hours.

**Drug**/Cell line	**Slope of linear regression curve**[a]		***p*-value**[b]
	Normoxia	*Hypoxia*	
**Doxorubicin**			
A673	-0.0115 ± 0.0008	-0.0089 ± 0.0004	*0.0079*
RDES	-0.0065 ± 0.0007	-0.0082 ± 0.0006	*0.0959*
SKES-1	-0.0097 ± 0.0007	-0.0102 ± 0.0007	*0.5808*
TC-32	-0.0075 ± 0.0004	-0.0089 ± 0.0007	*0.0954*
TTC-466	-0.0039 ± 0.0003	-0.0034 ± 0.0007	*0.6818*
**Vincristine**			
A673	-0.0123 ± 0.0004	-0.0110 ± 0.0003	*0.0422*
RDES	-0.0083 ± 0.0002	-0.0081 ± 0.0004	*0.6692*
SKES-1	-0.0093 ± 0.0006	-0.0077 ± 0.0018	*0.4214*
TC-32	-0.0076 ± 0.0007	-0.0085 ± 0.0008	*0.3863*
TTC-466	-0.0039 ± 0.0003	-0.0028 ± 0.0004	*0.0664*

[a]This is the slope of the graph of log CVC fluorescence *vs*. time. Proliferation of ESFT cells in the two environments was assessed by measuring the decrease in fluorescence of the CellVue®Claret (CVC) dye per cell at 0, 24, 48, and 72 hours and then plotting log-linear plots of CVC fluorescence against time. The slopes of these curves were then compared statistically by regression analysis and are shown above. Values shown are mean ± SEM of n = 6 determinations. [b]Slope in hypoxia is different from that in normoxia at *p*-value shown.

Table S2: Levels of apoptosis in ESFT cells incubated with doxorubicin and vincristine in normoxia and hypoxia for 48 hours.

Drug/Cell line	% Apoptotic cells[a]		p-value
	Normoxia	Hypoxia	
**Doxorubicin**			
A673	39.7 ± 3.1	66.3 ± 5.4	0.0043*
RDES	73.4 ± 3.6	88.1 ± 1.3	0.0022*
SKES-1	65.9 ± 6.9	80.6 ± 1.2	0.0931
TC-32	61.2 ± 2.2	60.0 ± 1.3	0.3939
TTC-466	45.2 ± 2.6	31.7 ± 2.2	0.0089*
**Vincristine**			
A673	79.1 ± 3.3	93.6 ± 2.4	0.015*
RDES	72.9 ± 1.2	91.9 ± 1.1	0.0022*
SKES-1	72.4 ± 2.8	89.5 ± 2.2	0.0022*
TC-32	50.4 ± 2.0	71.7 ± 2.2	0.0022*
TTC-466	47.1 ± 1.9	25.2 ± 1.5	0.0022*

[a]Apoptosis was assessed by Annexin-V/PI staining, acquired by FACS and analysed using CellquestPro™ software. The percentage value shown is the sum total of cells that stained positive for Annexin-V alone, Annexin-V + PI together, and PI alone for n = 6 determinations. The data were compared statistically using the non-parametric Mann-Whitney-Wilcoxon rank sum test. *Value in hypoxia different from that in normoxia at *p*-value indicated.
